# SARS‐CoV‐2, Influenza Virus, and Respiratory Syncytial Virus Coinfections Among Symptomatic Outpatients in Lorestan Province, Iran: A Retrospective Cross‐Sectional Study

**DOI:** 10.1002/hsr2.73242

**Published:** 2026-10-05

**Authors:** Ardalan Maleki Chegeni, Zahra Heydarifard, Gholam Reza Talei, Mohsen Mohammadi, Sayyad Khanizadeh

**Affiliations:** ^1^ Department of Virology, School of Medicine Lorestan University of Medical Sciences Khorramabad Lorestan Iran; ^2^ Hepatitis Research Center, Department of Virology, School of Medicine Lorestan University of Medical Sciences Khorramabad Lorestan Iran; ^3^ Hepatitis Research Center, Department of Pharmacognosy and Pharmaceutical Biotechnology, Faculty of Pharmacy Lorestan University of Medical Sciences Khorramabad Lorestan Iran

**Keywords:** acute respiratory tract infection, influenza virus, RSV, SARS‐CoV‐2, viral co‐infection

## Abstract

**Background and Aims:**

Pathogenic viruses represent a principal etiology of acute respiratory tract disease. Concurrent detection of multiple respiratory pathogens has been associated with diagnostic challenges, therapeutic complications, and unfavorable outcomes in patients with COVID‐19. This investigation aimed to characterize the frequency of concurrent SARS‐CoV‐2, influenza virus, and human respiratory syncytial virus (RSV) infections among individuals presenting with suspected COVID‐19 symptoms during the pandemic period in Lorestan Province, Iran.

**Methods:**

A retrospective observational study was conducted among 196 participants who presented with suspected COVID‐19 and clinically compatible respiratory symptoms at regional healthcare facilities in Lorestan Province from July through December 2022. Nasopharyngeal specimens were obtained from all participants, maintained at 2°C–8°C during transit on ice, and subsequently subjected to nucleic acid extraction and multiplex quantitative reverse‐transcription polymerase chain reaction (qRT‐PCR) analysis at the Molecular Virology Laboratory.

**Results:**

Of 196 participants evaluated, positivity rates were as follows: SARS‐CoV‐2, 40.81%; influenza virus, 30.10%; and RSV, 5.10%. Dual infections were identified in the following proportions: SARS‐CoV‐2 with RSV 3.57%, SARS‐CoV‐2 with influenza virus 11.22%, and RSV with influenza 3.06%. Simultaneous detection of all three pathogens was documented in 2.04% of the study population.

**Conclusion:**

This study documents the circulation of SARS‐CoV‐2, influenza virus, and RSV among symptomatic outpatients in Lorestan Province during the study period, including a limited number of dual and triple coinfections. These findings highlight the importance of continued respiratory virus surveillance and multiplex molecular testing in outpatient settings. Nevertheless, the small numbers of RSV‐positive patients, triple‐infection cases, and children younger than 6 years limit the strength of subgroup‐based inferences; therefore, age‐specific, pediatric, and symptom‐related findings should be interpreted cautiously. Further studies with larger and more diverse populations are warranted.

## 1 Background

Acute respiratory tract infections (ARTIs) constitute a substantial public health burden globally, characterized by considerable morbidity and mortality across all demographic populations [1]. These infections represent a leading reason for patient consultation at medical facilities and hospitalization, accounting for approximately 70% of respiratory illnesses affecting infants under 12 months of age. In developing nations, approximately 4–5 million pediatric deaths annually are attributed to ARTI complications. Young children under 5 years, geriatric populations, and immunocompromised individuals face heightened mortality risk from ARTI, positioning this population group among those with the most vulnerable health status. The clinical manifestations of these infections span the entire respiratory tract, ranging from mild rhinorrhea and cough to severe lower respiratory disease, including community‐acquired pneumonia [2].

Multiple infectious organisms, including viruses, bacteria, and parasites, contribute to the pathogenesis of ARTI. Viruses account for 30%–40% of infectious respiratory cases and represent the predominant causative agent of ARTIs. Principal viral pathogens associated with acute respiratory illness encompass human respiratory syncytial virus (RSV), human metapneumovirus (hMPV), influenza viruses type A and B (IFVA/B), human bocavirus (hBoV), human parainfluenza viruses (hPIV), rhinoviruses (RV), human coronaviruses (hCoV), and adenoviruses (AdVs). Respiratory tract disease may develop from infection with a single pathogen or result from concurrent infection with multiple infectious agents (viral or bacterial). Previous studies have suggested that polymicrobial respiratory infections may be associated with increased disease severity [3].

SARS‐CoV‐2 is classified as a positive‐sense single‐stranded RNA (ssRNA) virus within the family Coronaviridae, genus Betacoronavirus. Following initial identification in December 2019 in Wuhan, China, the pathogen demonstrated rapid international dissemination and was declared a global pandemic on March 11, 2020. As of December 8, 2024, the World Health Organization reported over 777 million confirmed cases and more than 7 million deaths worldwide [4, 5].

RSV, a negative‐sense single‐stranded RNA (ssRNA) virus from the family Pneumoviridae, is subdivided into subgroups A and B. This pathogen represents the leading infectious cause of ARTI, particularly in the pediatric population, especially children under 5 years of age. Clinical manifestations of pediatric RSV infection demonstrate considerable heterogeneity, ranging from self‐resolving upper respiratory tract symptoms to serious lower respiratory complications, including acute bronchiolitis, pneumonic infiltration, and hypoxemia. The health system and societal economic burden resulting from pediatric RSV disease are substantial [6].

The influenza virus genome comprises negative‐sense single‐stranded RNA (ssRNA), and the pathogen belongs to the family Orthomyxoviridae. Influenza virus serotypes A and B are recognized human respiratory pathogens. Type A influenza viruses possess pandemic and epidemic potential through their antigenic heterogeneity. As a zoonotic pathogen with broad host tropism affecting diverse animal species, influenza viruses maintain an ecological reservoir in avian species, from which human infections originate. Influenza virus activity in human populations typically peaks from September through May. Due to the capacity of certain influenza virus subtypes to cause severe respiratory disease in both avian and human populations, influenza remains designated as a clinically important respiratory pathogen [7, 8].

Accumulated evidence indicates that concurrent respiratory viral infections in hospitalized COVID‐19 patients may introduce diagnostic uncertainty, complicate therapeutic decision‐making, and adversely influence patient prognosis [4, 9]. Furthermore, simultaneous infection with multiple respiratory viruses frequently results in more pronounced clinical deterioration and elevated mortality rates [10]. Primary respiratory epithelial injury from certain viral pathogens may create favorable conditions for secondary SARS‐CoV‐2 acquisition and establishment of infection. During the COVID‐19 pandemic, concurrent SARS‐CoV‐2 detection with RSV or influenza has been documented across multiple studies, with reported prevalence rates between 0% and 22%. However, the clinical and epidemiological significance of such co‐infections within community and outpatient settings requires further clarification. All three viruses—influenza, SARS‐CoV‐2, and RSV—are transmitted via identical routes: direct contact, respiratory aerosol dispersion, and contamination of environmental surfaces. These pathogens demonstrate a broad clinical spectrum, from asymptomatic infection to severe systemic disease, manifesting as influenza‐like illness, respiratory pneumonia, and fatal outcomes [11, 12, 13]. This investigation aimed to determine the prevalence and epidemiological distribution of SARS‐CoV‐2, influenza virus, and RSV coinfections among symptomatic outpatients in Lorestan Province, Iran.

## 2 Materials and Methods

### 2.1 Patient Recruitment and Sample Collection

This retrospective cross‐sectional study was conducted between July and December 2022 in Lorestan Province, Iran. A total of 196 consecutive outpatients presenting to healthcare centers with symptoms suggestive of COVID‐19 or other ARTIs were enrolled. Eligible participants included individuals of all ages who presented with one or more respiratory symptoms, including fever, cough, sore throat, headache, body pain, lethargy, runny nose, or shortness of breath, and who underwent molecular testing for respiratory viruses. Immunocompromised individuals, transplant recipients, and patients receiving chemotherapy or active cancer treatment were excluded from the study.

Demographic variables (age, sex, and season of sample collection), clinical symptoms, and laboratory results were obtained from the participants' medical records and laboratory reports. Infection status was determined using multiplex real‐time RT‐PCR for SARS‐CoV‐2, influenza virus, and human RSV.

The study protocol was approved by the Ethics Committee of Lorestan University of Medical Sciences (Approval No. IR.LUMS.REC.1402.092), and written informed consent was obtained from all participants prior to enrollment.

Nasopharyngeal swab specimens were collected from each participant using sterile swabs. Specimens were placed in viral transport medium, stored at 2°C–8°C, and transported under cold‐chain conditions to the Molecular Laboratory, Department of Virology, Lorestan University of Medical Sciences, for viral RNA extraction and subsequent molecular analysis.

### 2.2 Variables

Demographic variables included age, sex, and season of sample collection. Clinical variables included fever, cough, sore throat, headache, body pain, lethargy, runny nose, and shortness of breath. Laboratory variables included the detection of SARS‐CoV‐2, influenza virus, and human RSV by multiplex real‐time RT‐PCR. Coinfection was defined as the simultaneous detection of two or more respiratory viruses in the same respiratory specimen.

### 2.3 Viral Nucleic Acid Extraction and Multiplex Real‐Time PCR

Viral ribonucleic acid (RNA) extraction was performed using the RNJia Virus Kit (ROJE Technologies, Iran) in accordance with the manufacturer's instructions.

Nasopharyngeal swab specimens were collected from all participants using sterile flocked swabs and immediately placed into viral transport medium (VTM). Samples were transported to the laboratory under cold‐chain conditions and processed according to standard laboratory procedures. Viral RNA was extracted from clinical specimens using the extraction protocol recommended by the manufacturer.

Detection of SARS‐CoV‐2, influenza virus, and human RSV was performed using the Geneova GA SARSFlu & RSV OneStep Real‐Time RT‐PCR Kit (Geneova Co., Iran), a multiplex real‐time reverse transcription polymerase chain reaction (RT‐PCR) assay designed for the simultaneous qualitative detection of these respiratory viruses. Amplification and fluorescence detection were carried out on a real‐time PCR instrument according to the manufacturer's instructions.

The assay simultaneously targets virus‐specific genomic regions of SARS‐CoV‐2, influenza virus, and RSV and includes an internal control to monitor the efficiency of RNA extraction, reverse transcription, and PCR amplification, as well as to detect potential PCR inhibition. Amplification results were interpreted according to the manufacturer's instructions. Samples with a cycle quantification Ct value of ≤ 40 for the corresponding target gene(s), together with valid internal and external controls, were considered positive.

Quality‐control procedures were implemented throughout the testing process. Each RT‐PCR run included the positive and negative controls provided with the kit to verify assay performance and to monitor for contamination. The internal control supplied with the assay was evaluated in every specimen to confirm successful nucleic acid extraction and amplification. Samples with invalid internal control results or inconclusive amplification curves were managed according to the manufacturer's recommendations.

### 2.4 Statistical Analysis

Statistical analysis was performed using SPSS software, version 22. No continuous variables were included in the final analyses. Categorical variables were summarized as frequencies and percentages. Prevalence estimates are presented with 95% confidence intervals where appropriate. For each viral outcome, including SARS‐CoV‐2 positivity, influenza positivity, RSV positivity, dual infections, and triple infections, comparisons were performed between participants with and without the specified infection outcome across age group, sex, season, and clinical symptom categories. Pearson's chi‐square test was used for comparisons when expected cell counts were adequate, and Fisher's exact test was applied when sparse cell counts were present. The main prevalence estimates were expressed as proportions; 95% CI were added for the principal overall prevalence measures where applicable. Because multiple subgroup comparisons were conducted, these analyses should be interpreted as exploratory, and the corresponding *p*‐values should be interpreted with caution. A two‐sided p‐value of less than 0.05 was considered statistically significant. No missing data were identified for the demographic, clinical, or laboratory variables included in the statistical analyses. All statistical tests were two‐sided.

Statistical reporting was performed in accordance with the recommendations of Assel et al. for reporting clinical research statistics [14].

## 3 Results

In this study, 196 respiratory samples were examined from individuals with respiratory symptoms who visited healthcare centers in Lorestan province during the summer and fall of 2022. The samples were investigated for the presence of RSV, SARS‐CoV‐2, and Influenza viruses. The study involved 196 samples, with 95 samples (46.49%) from males and 101 samples (53.51%) from females. Regarding the age distribution of the patients, 2 samples (1.02%) were under 6 years old, 11 samples (5.61%) were between 7 and 17 years old, 56 samples (28.57%) were between 18 and 35 years old, 105 samples (53.57%) were between 36 and 59 years old, and 22 samples (11.22%) were over 60 years old. The samples were collected during the summer and fall of 2022, with 98 samples (50%) collected in summer and 98 samples (50%) collected in fall [Table 1].

**Table 1:** Demographic and clinical characteristics of the study participants according to season.

Demographic data	Summer *N* (%)	Fall *N* (%)
Age group		
< 6	1 (1.02)	1 (1.02)
7–17	6 (6.12)	5 (5.10)
18–35	24 (24.48)	32 (32.65)
36–59	60 (61.22)	45 (45.92)
> 60	7 (7.14)	15 (15.31)
Sex		
Male	43 (43.9)	52 (53.0)
Female	55 (56.1)	46 (47.0)
Symptoms		
Fever	49 (50.0)	52 (53.06)
Cough	51 (52.04)	36 (36.73)
Sore throat	27 (27.55)	48 (48.97)
Nausea	5 (5.10)	8 (8.16)
Lethargy	45 (45.91)	42 (42.85)
Runny nose	4 (4.08)	28 (28.57)
Diarrhea	22 (22.44)	10 (10.20)
Headache	27 (27.55)	26 (26.53)
Shortness of breath	9 (9.18)	14 (14.28)
Body pain	40 (40.81)	29 (29.59)
Total	98 (100)	98 (100)

*Note:* Values are presented as *n* (%). Percentages were calculated within each season (Summer, *n* = 98; Fall, *n* = 98).

Out of the 196 samples, 80/196 cases (40.81%) tested positive for SARS‐CoV‐2, 59/196 cases (30.10%) tested positive for the influenza virus, and 10/196 cases (5.10%) tested positive for RSV. In this study, the results showed that out of 80 SARS‐CoV‐2 positive cases, 22 cases (27.5%) had coinfection with influenza infection, and 7 cases (8.75%) had coinfection with RSV infection. Among the 59 cases positive for the influenza virus, 22 cases (37.29%) had coinfection with SARS‐CoV‐2 infection, and 6 cases (10.17%) had coinfection with RSV infection. Additionally, among the 10 cases positive for RSV, 7 cases (70%) had coinfection with SARS‐CoV‐2 infection, and 6 cases (60%) had coinfection with influenza virus infection, and only 4 cases of triple infection were observed [Table 2].

**Table 2:** Detection of SARS‐CoV‐2, influenza virus, human respiratory syncytial virus (RSV), and viral coinfections among the study participants.

Virus infection	Positive cases *N* (%) [95% CI]	Negative cases *N* (%)
Influenza virus	59 (30.10) [24.11%–36.86%]	137 (69.90)
SARS‐CoV‐2	80 (40.81) [34.18%–47.81%]	116 (59.18)
RSV	10 (5.10) [2.79%–9.14%]	186 (94.90)
Mix: SARS‐CoV‐2 & influenza virus	22 (11.22) [7.53%–16.41%]	174 (88.78)
Mix: SARS‐CoV‐2 & RSV	7 (3.57) [1.74%–7.19%]	189 (96.43)
Mix: Influenza virus & RSV	6 (3.06) [1.41%–6.52%]	190 (96.94)
Triple Infection	4 (2.04) [0.80%–5.13%]	192 (97.96)

*Note:* Values are presented as *n* (%). Percentages were calculated using the total study population (*N* = 196).

Among the 101 samples from females, 37/101 cases (36.63%) tested positive for SARS‐CoV‐2, 27/101 cases (26.73%) tested positive for the influenza virus, and 4/101 cases (3.96%) tested positive for RSV. In the 95 samples from males, 43/95 cases (45.26%) tested positive for SARS‐CoV‐2, 32/95 cases (33.68%) tested positive for the influenza virus, and 6/95 cases (6.32%) tested positive for RSV. Because the number of participants in the < 6‐year age group was very small (*n* = 2), and the numbers of RSV‐positive (*n* = 10) and triple‐infection (*n* = 4) cases were limited, age‐specific findings should be interpreted with caution. Although RSV‐positive and coinfected cases were observed in younger age categories, these subgroup counts were too small for robust inference [Table 3].

**Table 3:** Association between viral infection status and demographic characteristics.

Characteristic (*n* per subgroup)	SARS‐CoV‐2 (*n* = 80)	*p*	Influenza virus (*n* = 59)	*p*	RSV(*n* = 10)	*p*	SARS‐CoV‐2 + Influenza virus (*n* = 22)	*p*	Influenza + RSV (*n* = 6)	*p*	SARS‐CoV‐2 + RSV (*n* = 7)	*p*	TripleInfection (*n* = 4)	*p*
AGE GROUP														
< 6 years (*n* = 2)	2 (2.5)	0.184	1 (1.7)	0.326	2 (20.0)	< 0.001*	1 (4.5)	0.141	1 (16.7)	< 0.001*	2 (28.6)	< 0.001*	1 (25.0)	< 0.001*
7–17 years (*n* = 11)	7 (8.8)	—	4 (6.8)	—	5 (50.0)	—	3 (13.6)	—	3 (50.0)	—	3 (42.9)	—	2 (50.0)	—
18–35 years (*n* = 56)	20 (25.0)	—	13 (22.0)	—	0 (0.0)	—	4 (18.2)	—	0 (0.0)	—	0 (0.0)	—	0 (0.0)	—
36–59 years (*n* = 105)	41 (51.3)	—	37 (62.7)	—	2 (20.0)	—	12 (54.5)	—	1 (16.7)	—	1 (14.3)	—	0 (0.0)	—
> 60 years (*n* = 22)	10 (12.5)	—	4 (6.8)	—	1 (10.0)	—	2 (9.1)	—	1 (16.7)	—	1 (14.3)	—	1 (25.0)	—
SEX														
Male (*n* = 95)	43 (53.8)	0.219	32 (54.2)	0.289	6 (60.0)	0.454	13 (59.1)	0.203	4 (66.7)	0.313	5 (71.4)	0.198	3 (75.0)	0.283
Female (*n* = 101)	37 (46.3)	—	27 (45.8)	—	4 (40.0)	—	9 (40.9)	—	2 (33.3)	—	2 (28.6)	—	1 (25.0)	—
Total positive	80 (40.8)		59 (30.1)		10 (5.1)		22 (11.2)		6 (3.1)		7 (3.6)		4 (2.0)	

* *p* < 0.05 was considered statistically significant. *p‐*values represent comparisons between participants with and without the specified infection outcome across the listed age‐group and sex categories, using chi‐square or Fisher's exact test as appropriate.

Out of the 98 samples collected in summer, 53 cases (54.08%) tested positive for SARS‐CoV‐2, 1 case (1.02%) tested positive for the influenza virus, and 2 cases (2.04%) tested positive for RSV. In the 98 samples collected in fall, 27 cases (27.55%) tested positive for SARS‐CoV‐2, 58 cases (59.18%) tested positive for the influenza virus, and 8 cases (8.16%) tested positive for RSV [Figure 1].

**Figure 1:**
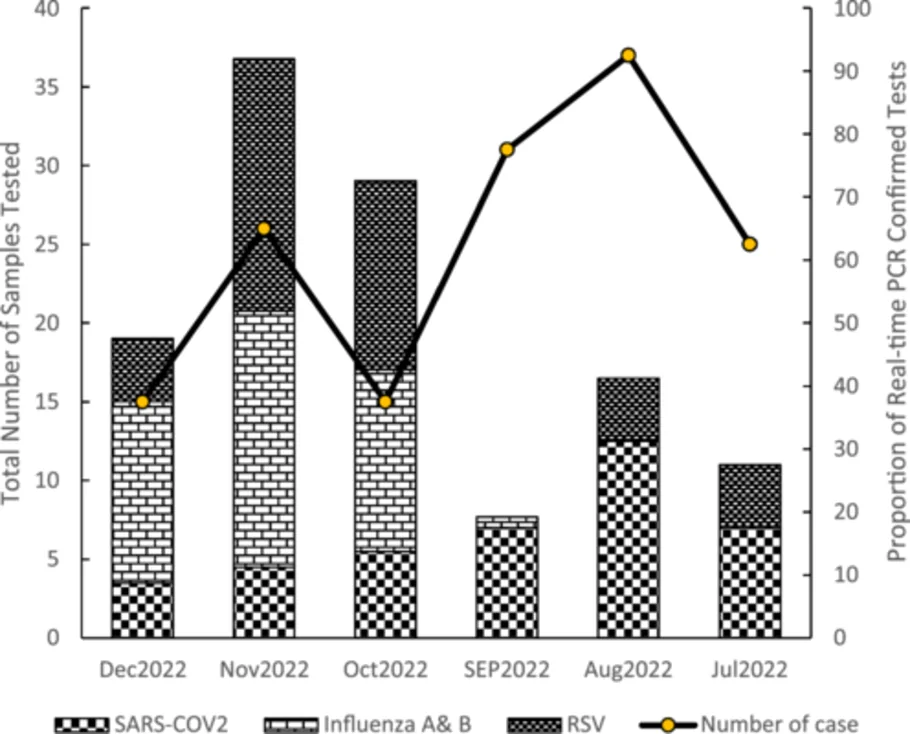
Seasonal distribution of SARS‐CoV‐2, influenza virus, and respiratory syncytial virus (RSV) detected among 196 symptomatic outpatients in Lorestan Province, Iran, between July and December 2022. Bars represent the number (or percentage, if applicable) of positive cases identified in summer and fall. SARS‐CoV‐2, severe acute respiratory syndrome coronavirus 2; RSV, respiratory syncytial virus.

In the conducted study, the relationship between viral infections and seasons was examined. SARS‐CoV‐2 was detected in 53/98 (54.08%) summer samples and 27/98 (27.55%) fall samples (*χ* ^2^ test, *p* < 0.001). Influenza virus was detected in 1/98 (1.02%) summer samples and 58/98 (59.18%) fall samples (*χ* ^2^ test, *p* < 0.001). SARS‐CoV‐2 was detected more frequently in summer (53/98, 54.08%), whereas the influenza virus was detected more frequently in fall (58/98, 59.18%). No statistically significant seasonal difference was observed for RSV detection. Additionally, regarding coinfections with the season of sample collection, the coinfection of RSV/influenza virus (*p* = 0.014) and Flu/SARS‐CoV‐2 (*p* = 0.0001) showed a significant correlation with the season (*p* = 0.014), occurring more frequently in fall compared to summer. However, coinfection of RSV/SARS‐CoV‐2 did not show a significant correlation with the season.

In the study, the prevalence of clinical symptoms in individuals positive for viral infections was also investigated. The most common clinical findings observed in these individuals included fever, cough, sore throat, fatigue, nasal discharge, diarrhea, headache, nausea, and shortness of breath, which are described separately for each viral infection in [Table 4]. However, associations involving RSV, dual infections, and triple infections should be interpreted cautiously because several of these outcome groups included very small numbers of positive cases.

**Table 4:** Association between viral infection status and clinical characteristics.

Clinical sign	SARS‐CoV‐2 (*n* = 80)	*p*	Influenza virus (*n* = 59)	*p*	RSV (*n* = 10)	*p*	SARS‐CoV‐2 + Influenza virus (*n* = 22)	*p*	Influenza virus + RSV (*n* = 6)	*p*	SARS‐CoV‐2 + RSV (*n* = 7)	*p*	TripleInfection (*n* = 4)
Fever	53 (66.3)	< 0.001*	35 (59.3)	0.101	8 (80.0)	0.061	18 (81.8)	0.002*	5 (83.3)	0.002*	7 (100.0)	0.009*	4 (100.0)
Cough	49 (61.3)	< 0.001*	28 (47.5)	0.340	6 (60.0)	0.243	9 (40.9)	0.445	3 (50.0)	0.547	5 (71.4)	0.141	2 (50.0)
Sore throat	33 (41.3)	0.286	34 (57.6)	< 0.001*	6 (60.0)	0.133	14 (63.6)	0.010*	3 (50.0)	0.547	4 (57.1)	0.254	2 (50.0)
Lethargy/Fatigue	46 (57.5)	0.003*	28 (47.5)	0.375	6 (60.0)	0.254	14 (63.6)	0.050*	4 (66.7)	0.250	5 (71.4)	0.147	3 (75.0)
Headache	53 (66.3)	0.005*	17 (28.8)	0.420	7 (70.0)	0.005*	10 (45.5)	0.039*	5 (83.3)	0.002*	6 (85.7)	0.002*	4 (100.0)
Body pain	44 (55.0)	< 0.001*	22 (37.3)	0.442	5 (50.0)	0.260	13 (59.1)	0.016*	4 (66.7)	0.122	4 (57.1)	0.208	3 (75.0)
Diarrhea	41 (51.3)	0.017*	37 (62.7)	0.186	2 (20.0)	0.508	12 (54.5)	0.124	1 (16.7)	0.254	1 (14.3)	0.680	0 (0.0)
Runny nose	13 (16.3)	0.571	20 (33.9)	< 0.001*	4 (40.0)	0.060	8 (36.4)	0.013*	3 (50.0)	0.056	3 (42.9)	0.087	2 (50.0)
Nausea	6 (7.5)	0.449	6 (10.2)	0.160	0 (0.0)	0.495	5 (22.7)	0.008*	0 (0.0)	0.001*	0 (0.0)	< 0.001*	0 (0.0)
Shortness of breath	14 (17.5)	0.033*	10 (16.9)	0.108	5 (50.0)	0.260	5 (22.7)	0.022*	3 (50.0)	0.095	4 (57.1)	0.459	2 (50.0)

* *p* < 0.05 was considered statistically significant. *p*‐values represent comparisons between participants with and without the specified infection category for each listed clinical symptom, using the chi‐square test or Fisher's exact test, as appropriate. *p‐*values were not calculated for the triple‐infection column because the sample size was very small (*n *= 4) and cell counts were insufficient for valid statistical testing.

## 4 Discussion

This retrospective study investigated the epidemiological distribution of SARS‐CoV‐2, influenza virus, and human RSV, as well as their coinfections, among symptomatic outpatients presenting to healthcare centers in Lorestan Province, Iran, during the summer and fall of 2022. SARS‐CoV‐2 was the most frequently detected virus, followed by the influenza virus and RSV. Coinfections involving these respiratory viruses were also identified, with SARS‐CoV‐2/influenza virus coinfection being the most common. These findings indicate that multiple respiratory viruses circulated concurrently during the COVID‐19 pandemic and suggest that viral coinfections were identified among patients presenting with acute respiratory symptoms.

The observed prevalence of SARS‐CoV‐2, influenza virus, and RSV generally agrees with reports from previous studies conducted during the COVID‐19 pandemic, although the reported frequencies vary considerably according to geographical location, study period, healthcare setting, and the characteristics of the study population. Similar investigations have documented concurrent circulation of these respiratory viruses during the pandemic. Differences in viral prevalence among studies may reflect variations in seasonal virus activity, diagnostic strategies, healthcare‐seeking behavior, and local epidemiological conditions [15, 16, 17].

In the present study, SARS‐CoV‐2/influenza coinfection was the most frequently observed dual infection, whereas coinfections involving RSV were less common. Comparable studies have also reported that coinfection with respiratory viruses occurs in a subset of patients with acute respiratory infections, although the reported frequencies differ substantially across populations. Such variation is likely attributable to differences in patient age, study design, inclusion criteria, circulation of respiratory viruses, and laboratory detection methods. Therefore, direct comparison of coinfection rates between studies should be interpreted with caution [16, 18].

Our findings showed seasonal differences in viral detection, with SARS‐CoV‐2 detected more frequently during the summer months and the influenza virus detected more frequently during the fall. These observations likely reflect the temporal pattern of virus circulation during the study period rather than typical seasonal epidemiology. Because this investigation was conducted over a limited 6‐month period at a single center, the findings should not be interpreted as representing long‐term seasonal trends [19, 20].

Several clinical symptoms, including fever, cough, headache, lethargy, and body pain, were commonly observed among virus‐positive patients [21]. Although some symptoms appeared more frequently among individuals with viral coinfections, the present study was not designed to compare clinical severity between patients with single and multiple viral infections. Furthermore, information regarding hospitalization, intensive care unit admission, oxygen requirement, or mortality was not available. Consequently, no conclusions can be drawn regarding the effect of viral coinfection on disease severity. Future prospective studies with larger sample sizes and comprehensive clinical outcome data are needed to clarify the clinical significance of respiratory viral coinfections [12, 22].

Comparison with previous reports indicates that RSV remained an important respiratory pathogen despite its relatively low detection frequency in our outpatient population. Similar SARS‐CoV‐2/RSV coinfection rates have been reported in surveillance studies from the United Kingdom [23], whereas higher frequencies have been observed among hospitalized pediatric populations in Brazil [24]. These differences most likely reflect variation in study populations, healthcare settings, age distribution, and periods of viral circulation. Likewise, previous investigations from Iran have reported unfavorable outcomes among hospitalized patients with respiratory viral coinfections. However, because our study included symptomatic outpatients, did not assess hospitalization, disease severity, or mortality, and identified only a small number of RSV‐positive and coinfected patients, comparisons regarding clinical outcomes should be interpreted cautiously [21, 24].

This study has several strengths. Multiplex real‐time RT‐PCR was used to simultaneously detect SARS‐CoV‐2, influenza virus, and RSV in all respiratory specimens, reducing the likelihood of diagnostic misclassification. In addition, all participants were recruited during the same surveillance period using identical laboratory methods, providing a consistent assessment of viral circulation within the study population.

Several limitations should also be acknowledged. This was a single‐center retrospective study with a relatively modest sample size, which may limit the generalizability of the findings. Clinical information regarding underlying comorbidities, influenza, and COVID‐19 vaccination status, previous respiratory infections, hospitalization, and disease outcomes was unavailable. Only three respiratory viruses were investigated, whereas other respiratory pathogens were not evaluated. The primary prespecified analyses included estimation of the prevalence of SARS‐CoV‐2, influenza virus, RSV, and viral coinfections. Subgroup analyses according to age, sex, season, and clinical symptoms were considered exploratory and should be interpreted cautiously. Therefore, statistically significant associations should be interpreted cautiously. Additionally, the small number of RSV‐positive patients (*n* = 10), triple infections (*n* = 4), and participants younger than 6 years (*n* = 2) limited subgroup analyses. Consequently, age‐specific patterns and symptom associations involving these subgroups should be interpreted with caution and require confirmation in larger multicenter studies.

Potential sources of bias include the retrospective study design, recruitment from a single outpatient setting, and the inclusion of only symptomatic individuals, which may limit the generalizability of the findings. In addition, selection bias cannot be excluded because asymptomatic infections and hospitalized patients were not included.

Overall, this study contributes to the epidemiological understanding of SARS‐CoV‐2, influenza virus, and RSV circulation among symptomatic outpatients during the COVID‐19 pandemic. Continued surveillance using comprehensive molecular diagnostic methods and larger multicenter studies is warranted to better characterize respiratory viral coinfections and their clinical significance.

## 5 Conclusion

The present study identified concurrent circulation of SARS‐CoV‐2, influenza virus, and RSV among symptomatic outpatients during the study period, with evidence of viral coinfections. Human RSV was detected in 3.06% of influenza‐associated coinfections and 3.57% of SARS‐CoV‐2‐associated coinfections, highlighting that although RSV was less frequently detected than SARS‐CoV‐2 and influenza virus, it remained an important circulating respiratory pathogen. These findings are consistent with surveillance studies from the United Kingdom, which reported a SARS‐CoV‐2/RSV coinfection rate of approximately 3.2% during the COVID‐19 pandemic. In contrast, higher coinfection rates have been reported among hospitalized pediatric patients in Brazil, likely reflecting differences in study population, clinical setting, and patient characteristics. However, because our study included only symptomatic outpatients and did not evaluate clinical severity, hospitalization, or mortality, direct comparisons of clinical outcomes should be interpreted with caution. Our findings primarily contribute to the epidemiological understanding of respiratory virus circulation and coinfections in the outpatient setting. Further multicenter studies with larger sample sizes and comprehensive clinical outcome data are needed to better define the clinical significance of respiratory viral coinfections and to inform evidence‐based prevention and management strategies.

## Author Contributions

**Ardalan Maleki Chegeni:** methodology, data curation, writing – original draft, writing – review and editing, visualization. **Zahra Heydarifard:** conceptualization, writing – review and editing, supervision, project administration, data curation. **Gholam Reza Talei:** validation, project administration. **Mohsen Mohammadi:** investigation. **Sayyad Khanizadeh:** data curation, supervision, visualization, writing – review and editing, conceptualization, project administration, formal analysis.

## Funding

The authors have nothing to report.

## Author Responsibility Statement

All authors have read and approved the final version of the manuscript. Dr. Sayyad Khanizadeh had full access to all of the data in this study and takes complete responsibility for the integrity of the data and the accuracy of the data analysis.

## Ethics Statement

The study was conducted in accordance with the Declaration of Helsinki and national and institutional standards. Written informed consent was obtained from all participants, who agreed to the use of their data for research purposes. For patients under the age of 16, written informed consent was provided by their parents or legal guardians, who also consented to the publication of the data. The Ethics Committee of Lorestan University of Medical Sciences approved this retrospective study under the approval code number IR. LUMS. REC.1402.092.

## Conflicts of Interest

The authors declare no conflicts of interest.

## AI Disclosure

The authors used ChatGPT (OpenAI, GPT‐5.5) and QuillBot Inc., online version to assist with English language editing and improvement of the manuscript. All scientific content, study design, data analysis, interpretation of the results, and final manuscript revisions were performed by the authors, who take full responsibility for the accuracy and integrity of the work.

## Transparency Statement

Dr. Sayyad Khanizadeh affirms that this manuscript is an honest, accurate, and transparent account of the study being reported; that no important aspects of the study have been omitted; and that any discrepancies from the study as planned (and, if relevant, registered) have been explained.

## Data Availability

The data that support the findings of this study are available on request from the corresponding author. The data are not publicly available due to privacy or ethical restrictions.
